# PROX1 restrains ferroptosis via SCD transcription activation in colorectal cancer: PROX1 inhibits ferroptosis in CRC

**DOI:** 10.3724/abbs.2023027

**Published:** 2023-02-23

**Authors:** Ruoxin Zhang, Dakui Luo, Zezhi Shan, Yufei Yang, Yi Qin, Qingguo Li, Xinxiang Li

**Affiliations:** 1 Department of Colorectal Surgery Fudan University Shanghai Cancer Center Shanghai 200032 China; 2 Department of Oncology Shanghai Medical College Fudan University Shanghai 200032 China; 3 Department of Pancreatic Surgery Fudan University Shanghai Cancer Center Shanghai 200032 China

Colorectal cancer (CRC) is one of the most commonly diagnosed cancers and the second most common cause of cancer-related mortality worldwide. Although several therapeutic approaches have been developed for CRC in recent years, the prognosis of patients who develop chemotherapy resistance remains poor.

Ferroptosis is an iron and lipid reactive oxygen species (ROS)-dependent form of programmed cell death, and it is distinct from other forms of regulatory cell death at multiple levels [1]. Ferroptosis is regulated by a complex network and is a research hotspot as a promising therapeutic target in human cancers.

Prospero homeobox 1 (PROX1) is an evolutionarily conserved transcription factor that controls the differentiation of different types of cells, including lymphatic endothelial cells, neuronal precursor cells, retinal progenitor cells, and hepatocytes [2]. PROX1 participates in cancer progression as both a tumor suppressor and an oncogene in different cancer types. *PROX1* is a novel target gene that is hypermethylated and transcriptionally silenced in primary and metastatic breast cancer [3]. PROX1 promotes dysplasia in colonic adenomas and CRC progression. It marks the transition from benign colon adenoma to carcinoma *in situ*, and its depletion inhibits the growth of human colorectal tumor xenograft models, while its transgenic overexpression promotes colorectal tumorigenesis. The potential interplay between PROX1 and ferroptosis in colon cancer remains unclear.

Stearoyl-CoA desaturase (SCD), an enzyme that catalyzes the rate-limiting step in monounsaturated fatty acid synthesis, has been reported to accelerate tumorigenesis [4]. Evidence indicated that SCD inhibited ferroptosis in cancer cells, and combination therapies of SCD1 inhibitors and ferroptosis inducers had a synergistic effect.

To explore the clinical significance of PROX1 expression in CRC, we detected PROX1 protein expression in both CRC ( *n*=276) and normal tissue ( *n*=135) specimens in the tissue microarray by IHC staining. The results demonstrated that PROX1 was upregulated in CRC tissues compared to that in normal tissues ( Figure 1A and Figure 1B and Supplementary Figure S1A). Moreover, high PROX1 expression was significantly associated with poor overall survival (OS) and disease-free survival (DFS) ( Figure 1B and Supplementary Figure S1B).

**Figure 1:**
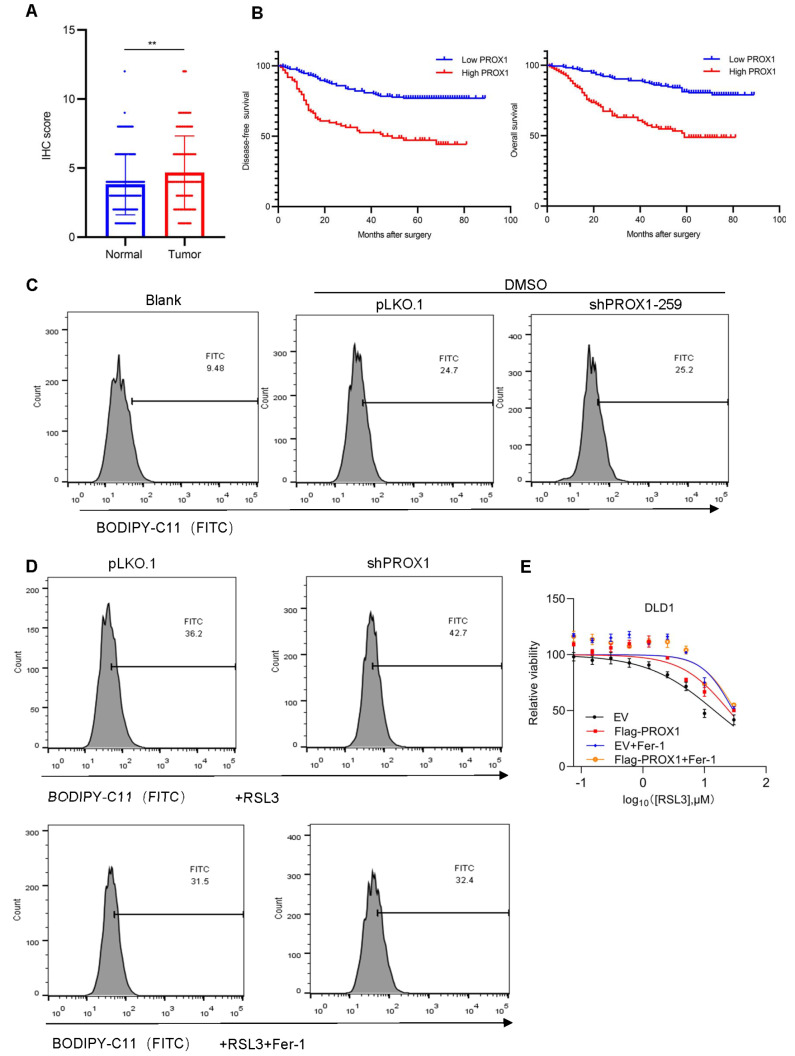
Figure 1 High expression of PROX1 inhibits lipid peroxidation and ferroptosis and indicates poor prognosis in CRC (A) PROX1 expression was upregulated in human CRC specimens compared to that in surrounding normal tissue specimens (** P<0.01). (B) High expression of PROX1 was associated with poor OS and DFS ( P<0.001). (C) Lipid ROS levels in the indicated DLD1 cells. (D) Lipid ROS levels in the indicated DLD1 cells treated with RSL3 or Fer-1. (E) Viability of the indicated DLD1 cells treated with RSL3 or Fer-1.

PROX1 induces CRC progression through diverse mechanisms. We further investigated whether PROX1 inhibits lipid peroxidation and ferroptosis to promote tumor growth and tumorigenesis. Bioinformatics analysis was performed using the ssGSEA algorithm in the TCGA cohort. We found that PROX1 expression was positively correlated with the ferroptosis score in colon cancer ( Figure 1B and Supplementary Figure S1C,D). To verify this correlation, a BODIPY 581/591C11 probe was used to detect oxidized lipids in DLD1 cells. Flow cytometric analysis showed that shRNA-mediated PROX1 knockdown did not affect lipid peroxidation levels under physiological conditions ( Figure 1C). Upon RSL3 (a ferroptosis inducer) treatment, downregulation of PROX1 caused an increase in lipid peroxidation levels. Notably, RSL3-induced lipid peroxidation could be rescued by ferrostatin-1, a ferroptosis inhibitor ( Figure 1D). Next, we determined the killing effect of RSL3 in DLD1 cells ectopically expressing PROX1. Overexpression of PROX1 impaired the killing effect of RSL3 in DLD1 cells, and RSL3-induced cell death could be rescued by ferrostatin-1 ( Figure 1E). Taken together, these data indicated that PROX1 protects CRC cells against ferroptosis.

To understand how PROX1 inhibits ferroptosis, five classic ferroptosis-related gene candidates ( *NFE2L2*, *STAT3*, *SCD*, *SLC7A11* and *GPX4*) were screened using RT-qPCR. *NFE2L2*, *STAT3* and *SCD* were significantly downregulated upon PROX1 depletion in DLD1 cells ( Figure 1B and Supplementary Figure S2A). We selected *NFE2L2* and *SCD* for further verification. Similarly, siRNA-based PROX1 downregulation inhibited *NFE2L2* and *SCD* mRNA expression levels in DLD1 cells ( Figure 2A). However, gain or loss of PROX1 resulted in a significant alteration of SCD protein level but not NFE2L2 protein level ( Figure 2B,C). Hence, we hypothesized that PROX1 might inhibit ferroptosis by upregulating the expression of SCD.

**Figure 2:**
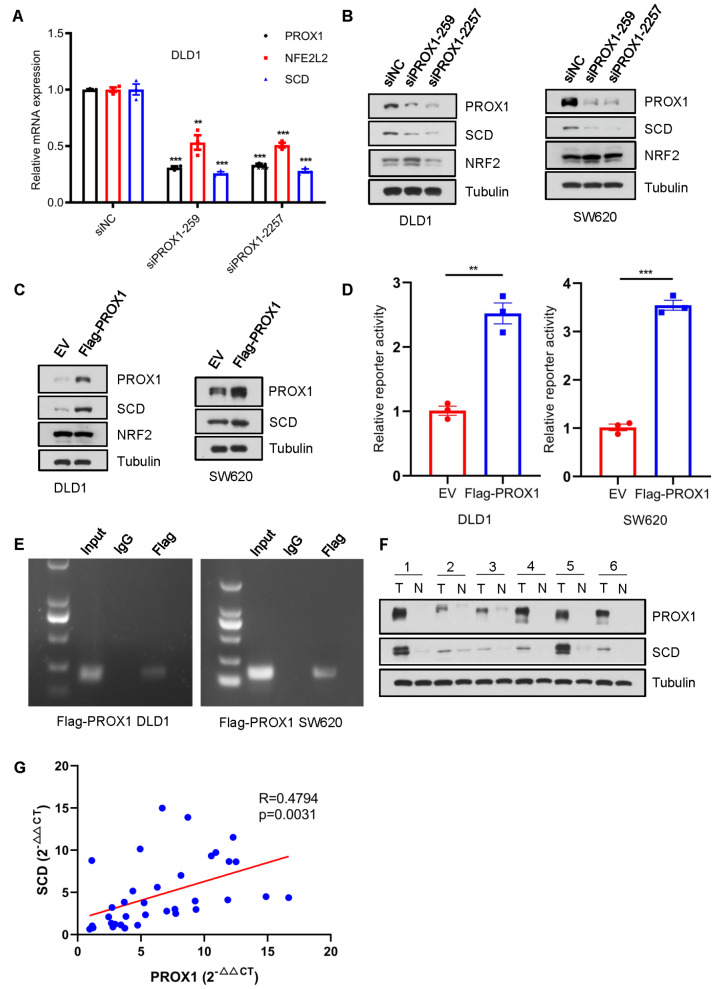
Figure 2 SCD is a putative target of PROX1 in CRC (A) Relative mRNA expression levels of PROX1, NFE2L2 and SCD were determined by qRT-PCR. (B) DLD1 cells and SW620 cells were transfected with the indicated siRNAs and subject to western blot analysis. Tubulin served as a loading control. (C) Western blot analysis in DLD1 cells and SW620 cells stably expressing ectopic PROX1 or empty vector. Tubulin was used as a loading control. (D) CRC cells stably expressing ectopic PROX1 or empty vector were transfected with a human PROX1 promoter-luciferase reporter, and then the cells were harvested for the luciferase reporter assay. (E) ChIP-PCR analysis of the SCD promoter region precipitated with anti-Flag antibody in CRC cells. Mouse IgG was used as a negative control. (F) PROX1 and SCD protein levels in six pairs of random CRC samples. N, adjacent normal specimens; T, matched tumor tissues. (G) PROX1 was positively correlated with SCD1 in CRC tissues derived from the FUSCC cohort. ** P<0.01, *** P<0.001.

Luciferase reporter assays revealed that overexpression of PROX1 activated the *SCD* promoter in both DLD1 and SW620 cells ( Figure 2D). Furthermore, direct binding of PROX1 to the *SCD* promoter was verified by chromatin immunoprecipitation (ChIP) assay in DLD1 and SW620 cells stably expressing Flag-tagged human PROX1 ( Figure 2E). Collectively, these results demonstrated that PROX1 can activate SCD transcription.

TCGA and GTEx data showed that PROX1 and SCD were both upregulated in CRC ( Supplementary Figure S2B). In addition, western blot analyses revealed that PROX1 and SCD proteins were upregulated in the tumor lysates compared to those in their normal counterparts ( Figure 2F). Correspondingly, the expression of PROX1 was positively correlated with SCD in CRC tissues derived from the TCGA cohort and the GSE39582 cohort ( Figure 1B and Supplementary Figure S2C,D). This correlation was further validated by qRT-PCR in 36 CRC patients from FUSCC ( Figure 2G).

Previous studies have indicated that PROX1 is implicated in the complex molecular mechanisms affecting the tumorigenesis of different organs. PROX1 can function either as an oncogene or as a tumor suppressor, depending on the cellular context. In CRC, PROX1 promotes tumor progression, and high PROX1 expression is associated with adverse characteristics and unfavorable patient outcomes [5]. PROX1 is a direct target of the Wnt/β-catenin signaling and is responsible for enforcing the stem cell phenotype and inhibiting differentiation by impairing the Notch pathway in CRC [6]. Moreover, PROX1 promotes metabolic adaptation and expansion of the CRC stem cell population to fuel tumor growth and metastases, highlighting the critical role of PROX1 in CRC tumorigenesis [ 7, 8] . Consistent with a previous study [6], we found that high expression of PROX1 was associated with poor prognosis in our cohort. Furthermore, PROX1 participated in the regulation of ferroptosis in CRC. Notably, CRC cells expressing high level of PROX1 are more resistant to ferroptosis, which may largely attenuate the potential therapeutic efficacy by inducing ferroptosis. Moreover, we demonstrated that PROX1 could activate SCD transcription. Although PROX1 contains a DNA-binding domain, it often serves as a co-regulator for transcription factors to regulate transcription [9]. Thus, how PROX1 activates SCD transcription remains unclear. Mass spectrometry can be used to identify the transcription factor that partners with PROX1 to regulate SCD transcription in the future. Similarly, a previous study reported that PROX1 activates HIF-1α transcription using luciferase reporter and ChIP assays. However, the authors did not clarify how PROX1 activates HIF-1α transcription [10].

In summary, we first demonstrated that PROX1 inhibited lipid peroxidation and ferroptosis in CRC cells after treatment with a ferroptosis inducer, which could be rescued by a ferroptosis inhibitor. To elucidate the underlying mechanism, we screened several classic target candidates that are critical for ferroptosis. Among these, we selected SCD for further study. Importantly, PROX1 could activate SCD expression by binding to its promoter. Our results suggested that the combination of SCD1 inhibitors and ferroptosis inducers may be a promising therapeutic strategy in PROX1-proficient CRC patients.

## Supporting information

22650Supplementary_Data

## Supplementary Data

Supplementary data is available at *Acta Biochimica et Biophysica Sinica* online.
