# Supplementary material for: PROX1 restrains ferroptosis via SCD transcription activation in colorectal cancer: PROX1 inhibits ferroptosis in CRC
Source: Acta Biochim Biophys Sin (Shanghai). 2023 Feb 23;55(4):691–4. doi: 10.3724/abbs.2023027 (PMC10195136; doi:10.3724/abbs.2023027)
Supplement: 22650Supplementary_Data [file 22650Supplementary_Data.pdf]

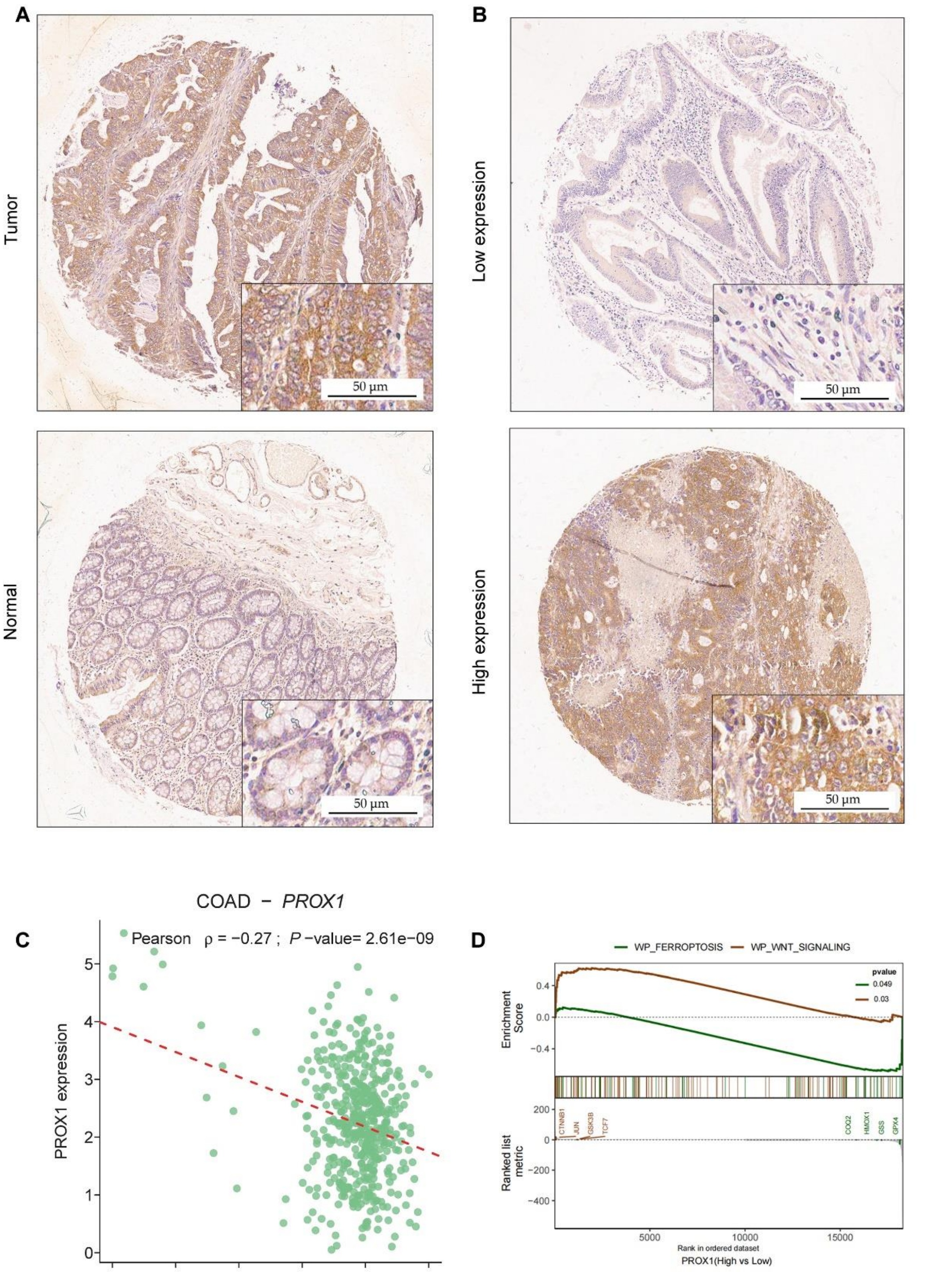

Supplementary Figure S1. Relationship between PROX1 and ferroptosis in CRC

(A) Representative IHC images of PROX1 expression in CRC and adjacent normal tissues. Scale bar: 50 μm. (B) Representative IHC images of high and low PROX1 expression in CRC tissues. Scale bar: 50 μm. (C) The Pearson's correlation analysis

revealed a negative correlation between PROX1 and ferroptosis ssGSEA score in TCGA database. (D) The GSEA demonstrates the relationship between PROX1 and ferroptosis in TCGA database.

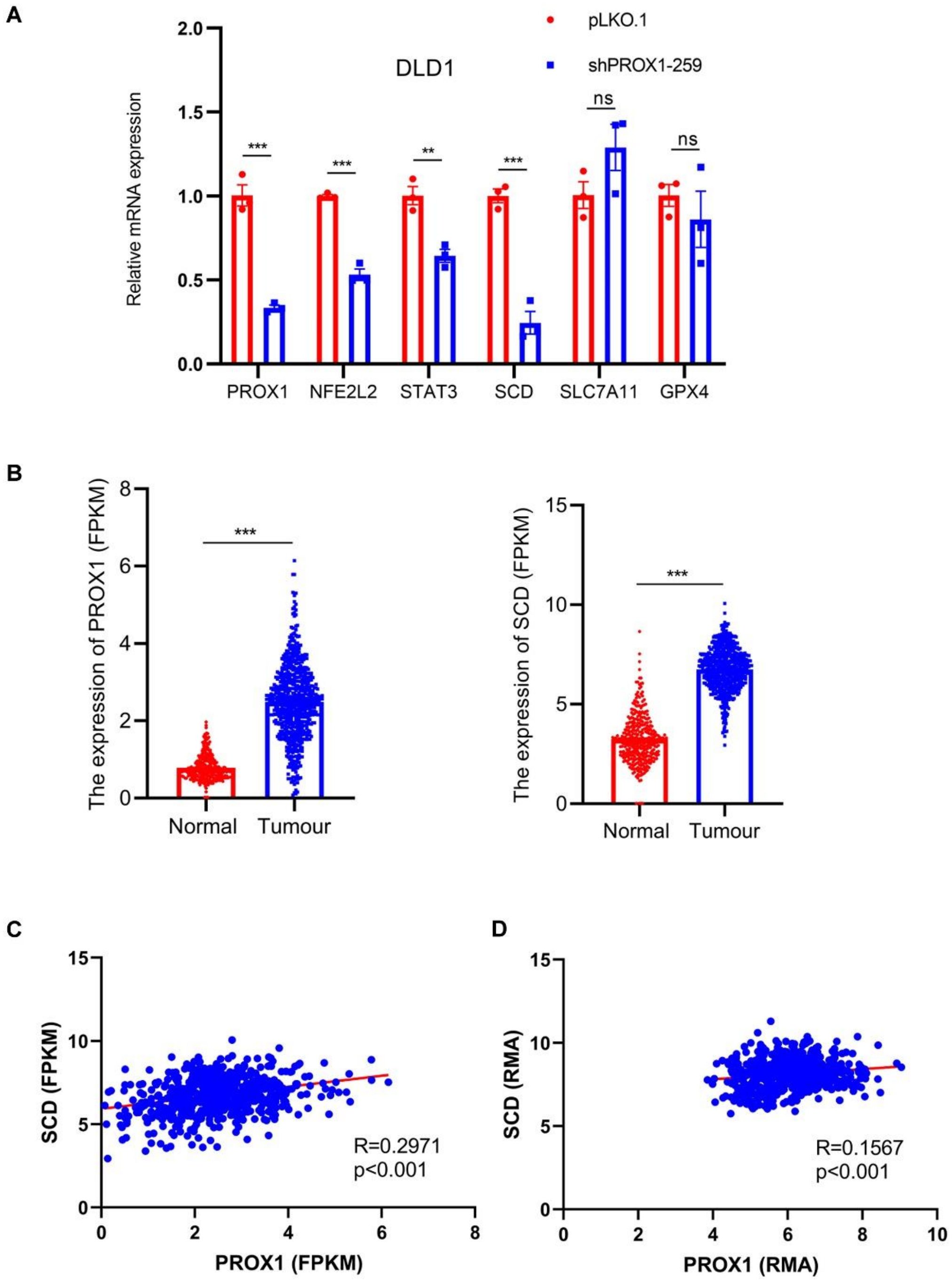

Supplementary Figure S2. Relationship between PROX1 and SCD in CRC (A) Relative mRNA expression levels of *PROX1*, *NFE2L2*, *STAT3*, *SCD*, *SLC7A11* and *GPX4* were determined by qRT-PCR. (B) PROX1 and SCD expressions were significantly higher in CRC tissues than those in normal tissues in the TCGA and GTEx datasets. (C) PROX1 was positively correlated with SCD1 in CRC tissues derived from TCGA dataset. (D) PROX1 was positively correlated with SCD1 in CRC tissues derived from GSE39582 dataset.
